# Decreased Cerebrospinal Fluid Antioxidative Capacity Is Related to Disease Severity and Progression in Early Multiple Sclerosis

**DOI:** 10.3390/biom11091264

**Published:** 2021-08-25

**Authors:** Margarete M. Voortman, Anna Damulina, Lukas Pirpamer, Daniela Pinter, Alexander Pichler, Christian Enzinger, Stefan Ropele, Gerhard Bachmaier, Juan-Jose Archelos, Gunther Marsche, Michael Khalil

**Affiliations:** 1 Department of Neurology, Medical University of Graz, 8036 Graz, Austria; margarete.voortman@medunigraz.at (M.M.V.); anna.damulina@medunigraz.at (A.D.); lukas.pirpamer@medunigraz.at (L.P.); daniela.pinter@medunigraz.at (D.P.); alexander.pichler@medunigraz.at (A.P.); chris.enzinger@medunigraz.at (C.E.); stefan.ropele@medunigraz.at (S.R.); juan.archelos@medunigraz.at (J.-J.A.); 2 Institute for Medical Informatics, Statistics and Documentation, Medical University of Graz, 8036 Graz, Austria; gerhard.bachmaier@medunigraz.at; 3 Division of Pharmacology, Otto Loewi Research Center, Medical University of Graz, 8036 Graz, Austria

**Keywords:** multiple sclerosis, cerebrospinal fluid, serum, antioxidant activity, magnetic resonance imaging

## Abstract

**Background:** Oxidative stress-induced neuronal damage in multiple sclerosis (MS) results from an imbalance between toxic free radicals and counteracting antioxidants, i.e., antioxidative capacity (AOC). The relation of AOC to outcome measures in MS still remains inconclusive. We aimed to compare AOC in cerebrospinal fluid (CSF) and serum between early MS and controls and assess its correlation with clinical/radiological measures. **Methods:** We determined AOC (ability of CSF and serum of patients to inhibit 2,2′-azobis(2-amidinopropane) dihydrochloride-induced oxidation of dihydrorhodamine) in clinically isolated syndrome (CIS)/early relapsing-remitting MS (RRMS) (*n* = 55/11) and non-inflammatory neurological controls (*n* = 67). MS patients underwent clinical follow-up (median, 4.5; IQR, 5.2 years) and brain MRI at 3 T (baseline/follow-up *n* = 47/34; median time interval, 3.5; IQR, 2.1 years) to determine subclinical disease activity. **Results:** CSF AOC was differently regulated among CIS, RRMS and controls (*p* = 0.031) and lower in RRMS vs. CIS (*p* = 0.020). Lower CSF AOC correlated with physical disability (*r* = −0.365, *p* = 0.004) and risk for future relapses (exp(β) = 0.929, *p* = 0.033). No correlations with MRI metrics were found. **Conclusion:** Decreased CSF AOC was associated with increased disability and clinical disease activity in MS. While our finding cannot prove causation, they should prompt further investigations into the role of AOC in the evolution of MS.

## 1. Introduction

Multiple sclerosis (MS) is a multifactorial, heterogeneous, chronic immune-mediated disease of the CNS, characterized by ongoing neuro-inflammation and -degeneration [1,2]. Converging evidence suggests that central features promoting the pathophysiology of MS are oxidative stress (OS) factors due to an imbalanced redox system [1,2,3,4,5,6,7,8].

Under normal conditions, oxidants are formed as a product of the aerobic cellular metabolism and exert specific intracellular functions [3,9,10]. Antioxidants, which are a wide variety of both enzymatic and non-enzymatic substances, counteract the potentially deleterious effects caused by oxidation of vital cellular components by these free radicals and related molecules. MS-specific disease characteristics, including activated immune cells, mitochondrial dysfunction and extracellular metal ion accumulation, may cause excessive release of reactive oxygen species (ROS) [1,2,3,4,5,6,7]. Further imbalance between ROS production and the body’s antioxidative defense causes OS and may contribute to the pathophysiology of MS through activation of inflammatory processes [11,12,13].

Numerous measures of ROS and antioxidants, i.e., single (metabolic) molecules, chemical elements, markers for lipid/total oxidation or antioxidant activity (of cells, tissues or fluids), have been investigated in the context of MS, predominantly in early phases of the disease (clinically isolated syndromes (CIS) and relapsing-remitting MS (RRMS)), but also in progressive stages [6,8,12,14,15,16,17,18,19]. Nevertheless, results are largely conflicting and the relation of OS parameters, i.e., (anti-)oxidative compounds, to clinical as well as subclinical measures of disease outcome remains unclear. Subclinical disease activity can be demonstrated by magnetic resonance imaging (MRI), which is of utmost importance in diagnosing as well as monitoring/surveillance of MS in clinical practice [20,21].

The cumulative activity of all antioxidants in body fluids of a patient is reflected by the total antioxidative capacity (AOC), which can be measured fluorometrically. We hypothesized that AOC may be affected in early MS and related to disease characteristics, including both clinical and subclinical measures of disease activity. Hence, we assessed AOC in cerebrospinal fluid (CSF) and serum in CIS and early RRMS compared to other neurological controls and probed its relation to longitudinal clinical and MRI data.

## 2. Subjects, Materials and Methods

This study was approved by the ethics committee of the Medical University of Graz, Austria (ethical approval number: 31-432 ex 18/19, 17-046 ex 05/06).

### 2.1. Patients and Controls

All participants included were seen at the MS outpatient clinic of the Department of Neurology, Medical University of Graz, from 2008 to 2013 and gave written and informed consent.

We included patients (*n* = 66, 66.7% female) meeting the following criteria: (1) diagnosis of CIS (*n* = 55) suggestive of MS or RRMS (*n* = 11), according to available criteria at time of inclusion [22,23]; (2) availability of a paired CSF and serum sample taken for diagnostic purposes; (3) no use of disease-modifying treatment prior to sampling (except for short-courses of corticosteroids); (4) available clinical follow-up data; (5) optionally available MRI examination of the brain at 3 Tesla at baseline and during follow-up.

The control group (*n* = 67, 67.2% female) consisted of subjects meeting the following criteria: (1) diagnosis of a neurological disease of non-inflammatory etiology (cranial/peripheral nerve palsy (*n* = 15, i.e., non-inflammatory neurological disease controls), headache (*n* = 29), or sensory disturbances (*n* = 23, i.e., symptomatic controls)) [24]; (2) availability of a diagnostic paired CSF and serum sample taken for diagnostic purposes; (3) routine-diagnostic variables measured in CSF and serum within normal range [24]; (4) no immunomodulatory or immunosuppressive treatment prior to sampling. Controls were matched to CIS/RRMS patients regarding sex and age.

### 2.2. Clinical Assessments and Follow-Up

Demographic and clinical data were recorded at time of diagnosis and during clinical follow-up (time since sampling median, 4.5 years; interquartile range (IQR), 5.2 years) in patients—age, sex, age at disease onset, time interval between the diagnosis of CIS and conversion to clinically definite MS (CDMS, upon second clinical relapse) and degree of disability as determined by the Expanded Disability Status Scale (EDSS) [25]. Upon sampling and diagnosis, scheduled follow-up examinations were performed by experienced neurologists.

Clinical relapses were recorded over time according to the following definition: at least one neurological symptom (re)appeared or an old symptom attributed to MS worsened for at least 24 h, succeeding a stable or improving neurological state during at least 30 days [23]. Upon confirmation of a clinical relapse during neurological examination, patients were usually treated with IV steroid pulses, for either 3- or 5-days, with 1000 mg/day methylprednisolone. Patients were considered to be in an active state of disease at the time of examination if sampling was performed within 30 days of a clinical relapse.

At the time of sampling, 19 patients (28.8%) had received corticosteroids within 30 days prior to CSF sampling and no one was on long-term disease-modifying treatments (DMTs). At some time during the clinical follow-up period, a total of 47 patients (71.2%) was prescribed DMTs. At the time of the last available clinical follow-up, 33 patients (50.0%) received the DMTs interferon beta (*n* = 16), glatiramer acetate (*n* = 8), dimethyl fumarate (*n* = 6), or fingolimod (*n* = 3). During clinical follow-up, 21 out of 55 CIS patients (38.2%) converted to CDMS, i.e., experienced a second clinical relapse.

### 2.3. Serum and CSF Sampling and Antioxidative Capacity Analyses

As part of a diagnostic evaluation, a total volume of 8 mL of peripheral blood was obtained and 6–10 mL of CSF was drawn by lumbar puncture in all subjects. Serum and CSF samples were aliquoted and stored at −80 °C immediately after routine diagnostic workup [26] until further analyses, according to international consensus guidelines [27].

The antioxidative capacity (AOC) of CSF and serum samples was determined by assessing the sample’s ability to inhibit 2,2′-azobis(2-amidinopropane) dihydrochloride (AAPH)-induced oxidation of dihydrorhodamine (DHR). Samples were pre-diluted 1:10 in phosphate buffered saline. AAPH, as well as the fluorescent DHR, were added to the assay buffer (20 mM HEPES, 150 mM NaCl, 10 g/L of Chelex-100, 1 mM AAPH, 7.5 μM DHR, pH of 7.4) in the absence or presence of the pre-diluted CSF (final dilution 1:300) or serum (final dilution 1:180) samples. Fluorescence intensity was measured. Readings (excitation wavelength, 485 nm; emission wavelength, 538 nm) were performed every 5 min for 60 min. Finally, the AOC per sample was calculated as percentage inhibition in fluorescence per minute due to oxidation of DHR after addition of the sample compared to blank assay buffer.

### 2.4. MRI Protocol

Imaging of the brain of CIS/RRMS patients was performed on a 3 Tesla Tim Trio scanner (Siemens Medical Systems, Erlangen, Germany) using a 12-element phased-array head coil. Structural imaging was performed using a T1-weighted three-dimensional (3D) Magnetization Prepared Rapid Acquisition Gradient Echo (MPRAGE) sequence (repetition time (TR)/echo time (TE)/inversion time (TI)/flip angle (FA) = 1.9 s/2.19 ms/0.9 s/9°; isotropic resolution = 1 mm) and a T2-weighted 2D fast Fluid Attenuated Inversion Recovery (FLAIR) sequence (TR/TE/TI = 9000 ms/70 ms/2500 ms, in plane resolution = 0.9 × 0.9 mm^2^, slice thickness = 3 mm).

Normalized regional brain tissue volumes of the caudate nucleus, globus pallidus, putamen and thalamus were determined using FSL-FIRST [28]. Normalized brain volumes were determined at baseline using SIENAX and longitudinal percentage of brain volume changes (PBVC) were assessed by applying SIENA at follow-up scans [29]. PBVC was annualized (PBVC/follow-up period).

For T2 hyperintense lesion load (T2LL) assessment, MS lesions were outlined with DispImage, a semi-automatic region growing technique that is based on local thresholding [30]. T2LL was calculated by multiplying the area of all lesion masks by the slice thickness. Image analyses were performed by an experienced neurologist, blinded to clinical data.

### 2.5. Statistical Analyses

Statistical analyses were performed using SPSS Statistics (version 25.0, IBM Corp. Armonk, NY, USA) and GraphPad Prism (version 5.00, GraphPad Software, San Diego, CA, USA). Normal distribution was tested for all variables using the Shapiro–Wilk test. We performed either the chi-square test for categorical data, or the independent t-test or the Mann–Whitney *U* test for dichotomous continuous or non-parametric data to determine group differences. Differences between paired samples were analyzed by the Wilcoxon signed-rank test or paired-samples sign test. Multiple comparisons were performed by using the Kruskal–Wallis test with subsequent post-hoc Dunn’s multiple comparison test. A univariate general linear model (GLM) was used to determine group differences with adjustment for covariates (demographic data and sample storage time). Correlation coefficients for AOC values with demographic, clinical and MRI data were assessed by Spearman (partial) correlations. Hierarchical linear and binary logistic regression analyses were performed for longitudinal data. The significance level was defined by *p* < 0.05 (2-tailed).

## 3. Results

### 3.1. AOC in Relation to Demographic and Laboratory Data

**Figure 1:**
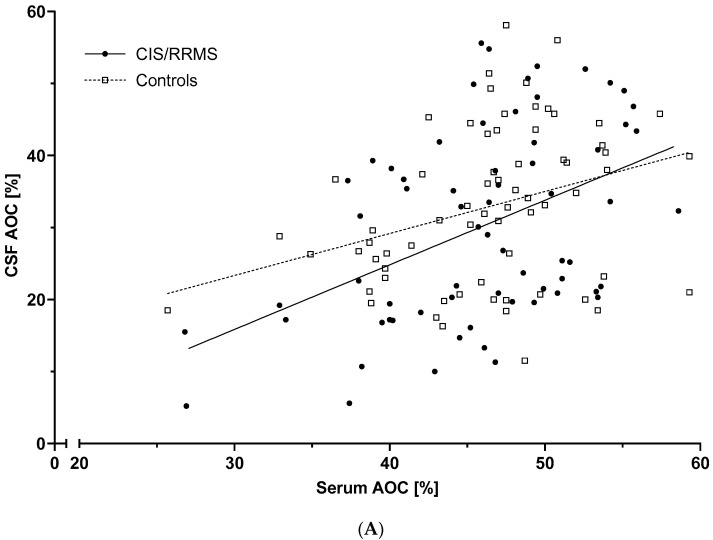

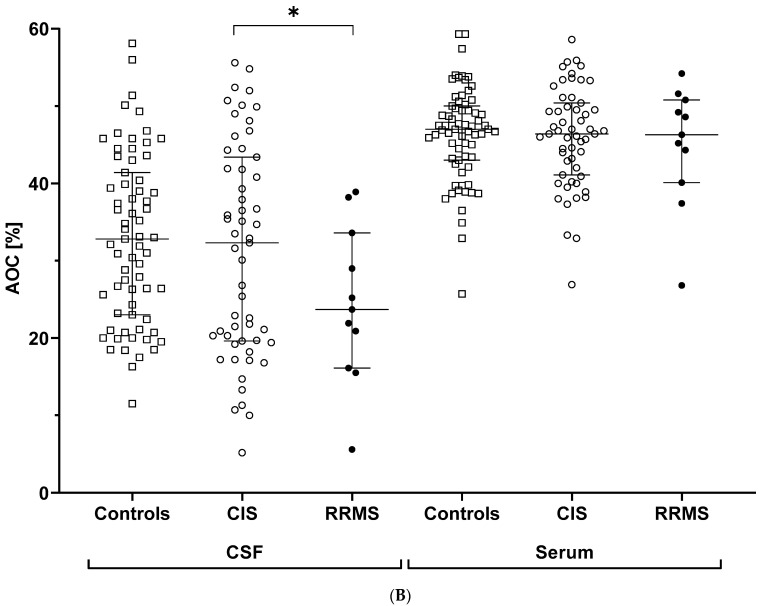
AOC in serum and CSF and its relation to disease course in MS. AOC values in serum and CSF in CIS and RRMS patients and controls. (**A**) CSF and serum AOC correlated mutually in both patients and controls (both *r* = 0.45, *p* < 0.001). (**B**) CSF AOC was significantly different between CIS and RRMS patients and controls (*p* = 0.031; adjusted for covariates) and showed lower values in RRMS compared to CIS patients (*p* = 0.020). Serum AOC was similar for CIS and RRMS patients and controls. AOC = antioxidative capacity; CIS = clinically isolated syndrome; CSF = cerebrospinal fluid; RRMS = relapsing-remitting multiple sclerosis. Horizontal lines represent median values. Significance (*p* < 0.05) was assessed between subgroups by univariate general linear model and Bonferroni post-hoc test. * *p* < 0.05.

Demographic and clinical data of patients and controls are given in Table 1. Routine diagnostic laboratory parameters, as well as AOC results, are listed in Table 2. In both patients and controls, AOC CSF and serum values were correlated to each other (both *r* = 0.45, *p* < 0.001; Figure 1A), with higher levels found in serum (both *p* < 0.001).

CSF AOC was not associated with sex and only a moderate linear correlation was found with age in controls (*r* = 0.382, *p* = 0.002). In serum, we found lower AOC in females compared to males (both in patients (median, IQR: female 44.9, 7.8%; male 51.4, 7.3%) and controls (median, IQR: female 45.2, 8.8%; male 49.7, 6.1%); *p* < 0.001); no association was found with age at sampling.

Serum but not CSF AOC was significantly lower in patients who used corticosteroids within 30 days prior to sampling (median, 44.1; IQR, 8.9%; *n* = 19) than that in those who did not (median, 47.3; IQR, 8.4%) (*p* = 0.020, corrected for multiple comparisons). No significant correlations were found between AOC levels and the time since the last corticosteroids were taken (median, 3; IQR, 6 days). AOC was not associated with the use of disease modifying treatments during follow-up.

### 3.2. CSF AOC in Association with Disease Severity in CIS and RRMS

AOC in CSF was differently regulated among CIS and RRMS patients and controls (*p* = 0.031; adjusted for age at sampling, sex and sample storage time). More specifically, CSF AOC in RRMS patients (estimated mean 26.2 ± SD 3.0%, adjusted for covariates) was lower than that in CIS patients (34.0 ± 1.4%) (*p* = 0.020, not corrected for multiple comparisons; Figure 1B).

**Figure 2:**
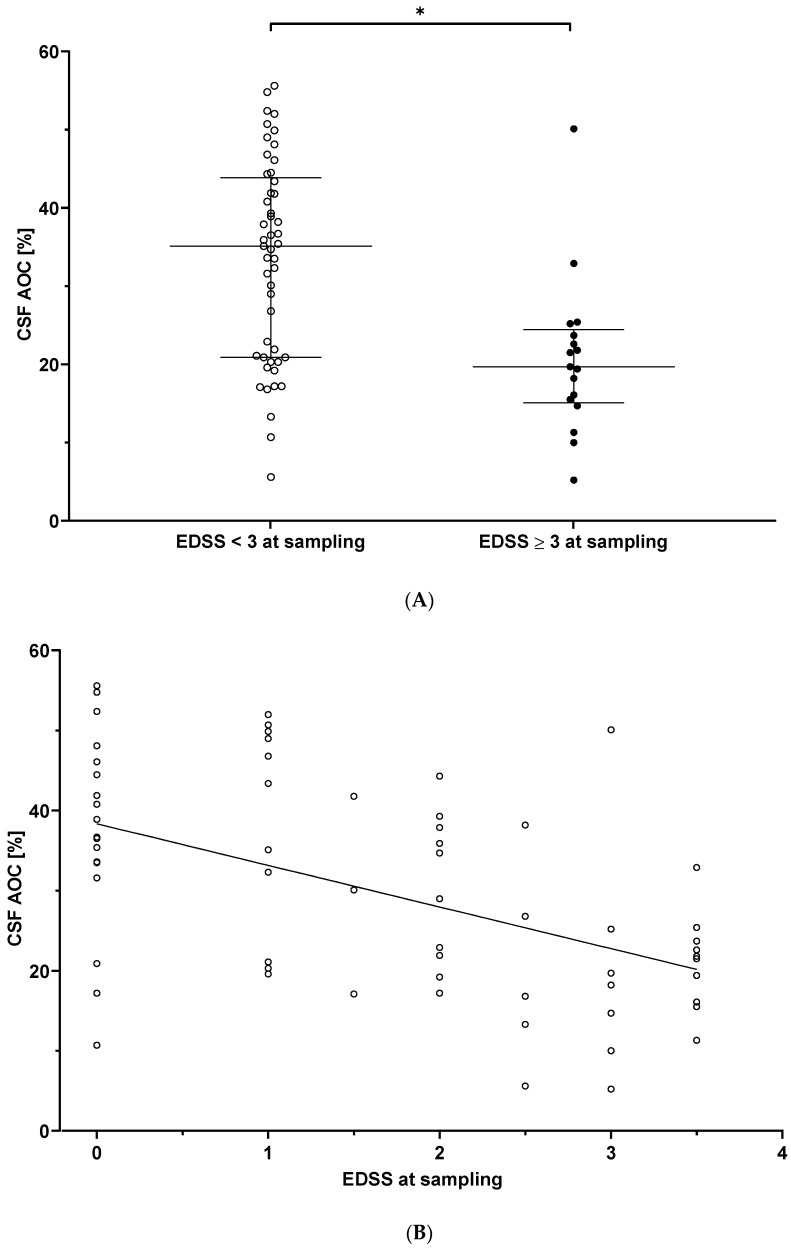

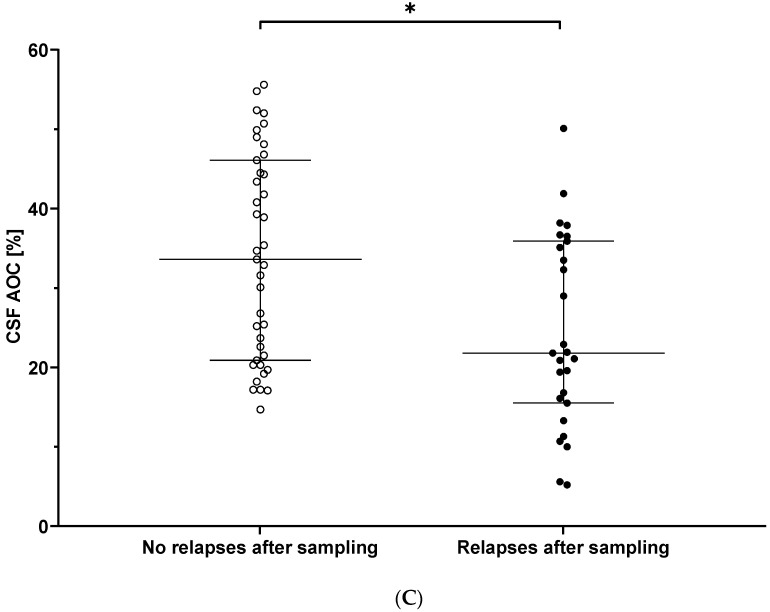
Decreased CSF AOC is associated with physical disability and predicts disease progression in MS. (**A**) CSF AOC was significantly lower in patients presenting with EDSS ≥ 3 (*n* = 17) those those with lower physical disability (*n* = 49) (*p* = 0.002). (**B**) CSF AOC correlated negatively with the EDSS at time of sampling (*n* = 66, *r* = −0.365, *p* = 0.004). (**C**) Graphical display of the association between CSF AOC and the occurrence of relapses after sampling. A decrease in CSF AOC was significantly associated with an increased likelihood of exhibiting new clinical relapses post sampling, as shown by binary logistic regression (exp(β) = 0.929, *p* = 0.033). During the follow-up time, 27 CIS/RRMS patients underwent a new clinical relapse and 39 did not. AOC = antioxidative capacity; CIS = clinically isolated syndrome; CSF = cerebrospinal fluid; EDSS = Expanded Disability Status Scale; RRMS = relapsing-remitting multiple sclerosis. Significance (*p* < 0.05) was assessed by Mann–Whitney *U* test, partial Spearman correlation (covariates age at sampling, sex, sample storage time, time between sampling and EDSS examination), or binary logistic regression. * *p* < 0.05.

Lower CSF AOC levels were associated with increased disability at time of sampling. Patients with higher EDSS scores (≥3; *n* = 17) had significantly lower CSF AOC (estimated mean 26.0 ± 2.6 %, adjusted for covariates) compared to those with lower EDSS scores (<3; *n* = 49, CSF AOC 34.6 ± 1.4%) (*p* = 0.022; Figure 2A). CSF AOC also negatively correlated with EDSS at time of sampling (all patients, *r* = −0.365, *p* = 0.004) (Figure 2B).

### 3.3. CSF AOC and Prediction of Clinical Disease Activity over Follow-Up

Binary logistic regression ascertained that a decrease in CSF AOC upon sampling was associated with an increased likelihood of exhibiting new clinical relapses (exp(β) = 0.929, *p* = 0.033) (Figure 2C). The model (*p* = 0.002; including demographic (age/sex) and time dependent covariates (sample storage time, follow-up time) and CSF AOC) explained 33.4% (Nagelkerke *R^2^*) of variance for cases with future relapses (*n* = 27) and classified 77.4% correctly. We did not find any association of AOC with the time from sampling until the next relapse (median, 1.5; IQR, 3.30 years) or annualized relapse rate at follow-up in RRMS (*n* = 32).

### 3.4. Serum AOC and the Association with Clinical Disease Characteristics

AOC in serum did not differ between patient and control subgroups (Figure 1B). Serum AOC was not associated with any of the disease specific parameters, i.e., physical disability determined by the EDSS, clinical disease activity (confirmed relapses) at time of sampling or during follow-up.

### 3.5. AOC in Relation to MRI Metrics

One or more MRI examination(s) was/were available in 64 (97.0%) of all patients (time interval of first scan since sampling median, 6.3; IQR, 10.6 months). We included baseline scans acquired within 1 year from serum/CSF sampling (*n* = 47) and follow-up scans within 2–5 years upon sampling (*n* = 34; time interval since sampling median, 3.5; IQR, 2.1 years). Normalized brain volumes were assessed for the entire brain, grey and white matter, cortical regions and ventricles. Global brain atrophy was examined by the PBVC and its annualized rate accounting for differences in follow-up and the T2LL was assessed at baseline and follow-up (Table 3). AOC of CSF and serum were not associated with any of the MRI metrics, neither at baseline nor at follow-up, nor did further sub-analyses (including less variable time intervals from sampling to MRI scan) yield significant correlations.

## 4. Discussion

Oxidative stress is believed to play a central role in MS pathophysiology. The deleterious effects of reactive oxygen species leading to tissue damage are counteracted and, therefore, also in part determined by the body’s ability to delay or prevent oxidation, which is subsumed under the term total antioxidative capacity (AOC). We here provide results indicating that the AOC in the CSF may be reduced in MS patients and relates to physical disability determined by the EDSS. Lower CSF AOC levels further seemed to partly predict the development of clinical relapses.

Oxidation–reduction (redox) status is an important balance between reactions causing deleterious and counteracting effects and is regulated in the body through highly complex mechanisms. The body’s redox system comprises of numerous both oxidative and antioxidative compounds, of which many still remain unknown or hard (or even impossible) to measure [5,11,31]. An antioxidant has been defined as “any substance that, when present at low concentrations compared to those of an oxidizable substrate, significantly delays or prevents oxidation of that substrate” [32] and can be classified as either enzymatic (e.g., superoxide dismutase and catalase) or non-enzymatic (low molecular weight elements, e.g., uric acid, albumin, vitamins and antioxidant ions) [3,12,13]. The protective relevance of a specific antioxidant depends on the type of ROS generated (e.g., superoxide, hydrogen peroxide, hydroxyl and peroxyl radicals), the target of damage and where and how it was produced [32]. Given the above-mentioned points, apparently, interpretation and comparison of studies investigating single redox parameters, also in the light of the markedly heterogeneity of MS, is limited [5]. Therefore, a representation of the in vivo balance of the redox system by the AOC, cumulatively for all (anti)oxidants, may be advantageous compared to measuring single compounds [11,31,33]. So far, it remained inconclusive whether the AOC is altered in the CSF and/or serum in MS and if its levels are related to clinical and imaging parameters.

We found a reduced AOC in the CSF of patients with RRMS vs. CIS, suggesting a relatively decreasing capability of defense mechanisms against OS as the disease advances. This is in line with two recent studies showing decreased CSF AOC in patients with RRMS (*n* = 22 [34] and *n* = 57 [35], respectively) compared to controls (*n* = 20 in both studies). The latter study also found CSF AOC in RRMS to be decreased compared to CIS patients (*n* = 50), who in turn also had lower AOC levels compared to controls. Notably, both previous studies included patients that were older and had a longer disease duration, as well as more severe physical disability, than our patients, possibly explaining unconforming results. The finding of decreased AOC in CSF in early MS is intriguing and may reflect the predominant role of OS already early in the disease, which can emerge either through the accumulation of (excessively released) ROS, or a depletion of (the activity of) antioxidants [12,13]. AOC can be altered under the influence of several excessive redox dependent changes relevant in MS, such as mitochondrial failure, high lipid peroxidation, loss of BBB integrity, hyperactivation of oxidative compounds, or intake of dietary antioxidants [11,35,36].

Independent from disease stage, we found lower CSF AOC levels to be associated with higher EDSS scores as measure of physical disability at time of sampling. It would be likely that accrual in physical disability is provoked by the perturbation of the body’s redox potential in the CNS. A recent study described higher CSF AOC in patients with lower vs. higher EDSS scores (≤3 vs. >3 in CIS, ≤5 vs. >5 in RRMS), with a moderately to strongly negative correlation between CSF AOC and EDSS [35]. Another study showed a similar relation, although non-significant, between CSF AOC and EDSS, but with accompanying positively significant correlations of CSF AOC with the antioxidative marker Klotho and, in turn, Klotho with EDSS [34]. Analogous correlations between physical disability and other specific antioxidative markers catalase and superoxide dismutase have been reported [15]. Our data strengthen the hypothesis that perturbation of the defense mechanisms against OS may promote ongoing tissue damage and neuroaxonal loss—the latter representing the pathophysiological substrate of permanent disability—making the AOC a potential treatment target.

The longitudinal clinical data presented here, including a relatively long follow-up time ranging from 2 to 7 years, further show a significant relationship of lower CSF AOC levels at baseline with an increased risk of developing new relapses over time. A few preceding studies also reported decreased AOC parameters either during or as precursor to (clinical) relapses [5,14,18], confirming that a weakened CSF AOC could unfavorably impact the long-term clinical course in MS.

Serum AOC appeared to be decreased in patients with the use of corticosteroids prior to sampling, although this effect was marginal and results in this subgroup were still similar to the controls. Besides, AOC was not affected by the duration of the therapy, nor was the CSF AOC affected by corticosteroid usage. A previous study also found no effect of corticosteroid usage on various markers of oxidative stress (oxidants and antioxidants, including total AOC) in serum and saliva [19]. Only beneficial effects on relieving oxidative stress in MS by the use of corticosteroids have been described, e.g., by a decrease in the lipid peroxidation [37] and oxidative and nitrosative stress markers in CSF/serum of patients [38]. From this, we do not expect corticosteroids to exert a significant influence on results of AOC.

For serum AOC, no associations with other demographic, clinical and morphological data were found, which is in line with findings of other recent studies [8,31,39,40]. However, few other reports showed reduced serum or plasmatic AOC in MS, compared to healthy controls. Importantly, the patient cohorts in these studies differed from ours, as these included more advanced MS with longer disease duration, mostly DMT-treated RRMS [35,41,42,43,44,45], progressive forms of MS (primary or secondary) [11,40], or a combination thereof [14,18,19,46,47,48,49]. Only one study including subjects with seemingly similar demographic and clinical features to ours demonstrated serum AOC to be decreased in CIS and even more in CDMS patients, compared to controls (all groups *n* = 49). Besides, a decrease in serum AOC was associated with an increased risk in CIS patients to convert to CDMS (i.e., to suffer from a second clinical attack, *n* = 25) during a 3-year follow-up time [14]. Future studies including larger sample sizes should clarify if serum AOC may be used as biomarker for disease severity and progression and whether alterations in serum AOC only appear in more advanced MS.

In an attempt to show a relation of AOC with subclinical disease activity, we calculated correlations with cerebral MRI metrics, including T2 lesion load and normalized brain volumes; however, these were not significant. Increasing lesion volume and atrophy are markers of subclinical disease activity and can be used as surrogates to determine treatment response [50,51], but these correlations come from controlled and adequately powered trials and most probably correlations are stage-dependent in “real-life” settings. Importantly, our study was not primarily designed to find a relationship with MRI measures; hence, the number of scans included was limited and the time between sampling and MRI scans varied over our cohort. Additionally, all patients included had a relatively short disease duration accompanied by only minor morphologic brain changes. The resulting potential to find significant associations between AOC and MRI metrics thus remains quite limited for several reasons. Only one recent study reported lower CSF AOC to be associated with higher T2 lesion number (number of lesions ≤9 vs. >9 in CIS, or ≤40 vs. >40 in RRMS, respectively), although no significant correlations with MRI metrics (absolute number of T2-weighted lesions and volume of gadolinium-enhanced lesions) were found [35]. Another study reported a positive correlation between serum AOC and T2 and gadolinium-positive lesion numbers, although this was only seen in interferon-beta-treated RRMS patients in which lesion numbers were in part also correlated to the patients’ disease duration [40]. Further studies including a higher number of patients, more advanced MS disease courses and more frequent MRI scans at regular intervals aiming to capture subclinical disease activity as in phase II treatment trials are needed to draw firm conclusions on the relation of AOC to MRI metrics.

It is important to note that direct comparisons of different studies on AOC may be limited, as different analysis methods have been applied, either proposed to express the total antioxidant level or the antioxidative capacity [52]. Some assays do not measure important antioxidants adequately/efficiently, while, with others, it is unclear which antioxidants contribute to what extent to its values, which may confound results [12,18,45,52]. 2,2′-Azobis(2-amidinopropane) dihydrochloride (AAPH) is a free radical initiator commonly used to evaluate the antioxidant capacity of biological fluids (e.g., CSF and serum). We determined the AOC of CSF and serum samples by assessing the ability of the samples to inhibit the oxidation of dihydrorhodamine (DHR) induced by AAPH. DHR exposed to AAPH oxidizes and begins to fluoresce (at a linear rate). The rate of oxidation of DHR is significantly lower when CSF (or serum) is added. Harmonization of different analysis methods is of great importance to further study this marker in different MS cohorts.

Some limitations of the current study need to be acknowledged specifically. A relatively low number of patients was included, especially in the RRMS group. The focus of our study was further on early disease, which did not include patients with progressive disease and, thereby, not the entire MS spectrum was covered. As patients with CIS and early RRMS are still scarcely represented in the literature, we nonetheless strongly believe that our study contributes to the better understanding of redox imbalance in the pathophysiology during disease onset and early disease. Nevertheless, it would be of interest to include patients with prolonged disease duration and/or more progressed disease, in particular, since some previous studies did find disease-specific associations with the AOC both in CSF and serum that we did not.

Altogether, we here indicate that decreased CSF AOC is associated with increased disease activity and progression in CIS and early RRMS. The AOC therewith seems to be a useful factor to target in order to counteract MS pathology already in the earliest phases of the disease. Our study provides promising results that could serve as a good basis for future research on extended cohorts to further elucidate the clinical significance of alterations in the AOC in MS. More comprehensive MRI data, as well as cognitive analyses, should be included to investigate the potential role of the AOC as treatment target, or its contribution as a prognostic tool.

## Figures and Tables

**Table 1:** Demographic and clinical data of study subjects.

	CIS/RRMS *n* = 55/*n* = 11	Controls *n* = 67
*n* female	44 (66.7)	45 (67.2)
Age (years)	32.0 (26.4–39.1)	32.7 (25.2–44.9)
Age disease onset (years)	31.1 (25.2–39.0)	N/A
Disease duration (months)	0.5 (0.3–3.9)	N/A
Clinical FU (years)	4.5 (1.9–7.0)	N/A
EDSS	1.5 (0.0–3.0)	N/A
EDSS (in remission)	1.0 (0.0–2.0)	N/A
EDSS last FU (in remission)	0.0 (0.0–1.5)	N/A
*n* Active disease ≤ 30 days prior to sampling	45 (68.2)	N/A
*n* Cortisone ≤ 30 days prior to sampling	19 (28.8)	N/A
*n* DMT	0 (0)	N/A
*n* DMT at last FU	33 (50.0)	N/A
ARR last FU (*n* = 21/11) ^†^	0.5 (0.3–0.9)	N/A

Unless otherwise indicated, data are given for time at sampling. Values are given as number (%) or as median (25th–75th quartile). Differences between CIS/RRMS and controls regarding sex (*p* = 0.951) and age (*p* = 0.589) were not significant. ARR = annualized relapse rate; CIS = clinically isolated syndrome; DMT = disease modifying treatment; EDSS = Expanded Disability Status Scale; FU = follow-up; *n* = number of subjects; N/A = not applicable; RRMS = relapsing-remitting multiple sclerosis. ^†^ ARR for RRMS at follow-up, disease duration ≥ 1 year.

**Table 2:** Routine diagnostic parameters and AOC in CSF and serum of patients and controls.

	CIS/RRMS *n* = 55/*n* = 11	Controls *n* = 67	*p*-Value
CSF white cell count (nr/µL) (ref. ≤ 4)	9 (5–17)	1 (1–2)	<0.001 ^a^
*n* OCB positive	64 (97.0)	0 (0)	<0.001 ^b^
Q_alb_ (×10^3^)	4.89 (4.19–6.96)	4.96 (4.11–5.84)	0.434 ^a^
*n* increased Q_alb_ (BBB disruption)	17 (25.8)	0 (0)	<0.001 ^b^
CSF lactate (mmol/L) (normal range < 2.1)	1.5 (1.4–1.7)	1.4 (1.4–1.5)	0.027 ^a^
CSF total protein (mg/dL) (normal range < 45)	35 (27–43)	30 (27–35)	0.012 ^a^
CSF AOC (%)	29.5 (19.6–40.8)	32.8 (23.0–41.4)	0.180 ^a^
Serum AOC (%)	46.4 (41.1–50.4)	47.0 (43.0–50.0)	0.763 ^a^

Data are given for time at sampling. Values are given as number (%) or as median (25th–75th quartile). Significance (*p* < 0.05) was assessed between subgroups by Mann–Whitney *U* test ^a^ or chi-squared test ^b^. AOC = antioxidative capacity; BBB = blood–brain barrier; CIS = clinically isolated syndrome; CSF = cerebrospinal fluid; *n* = number of subjects; OCB = oligoclonal bands; Q_alb_ = CSF/serum albumin quotient; RRMS = relapsing-remitting multiple sclerosis.

**Table 3:** MRI metrics of MS patients at baseline and follow-up.

	MRI BL *n* = 47	MRI FU *n* = 34	*p*-Value *n* = 22
Time from MRI to body fluid sampling ^a^ (months), ^b^ (years)	5.06 (1.22–7.52) ^a^	3.51 (2.53–4.60) ^b^	<0.001
Normalized brain volume (cm^3^)	1617.9 (1566.1–1662.7)	1582.8 (1536.7–1623.2)	0.001
Normalized grey matter volume (cm^3^)	832.6 (786.1–861.4)	798.4 (772.0–831.8)	0.004
Normalized white matter volume (cm^3^)	779.9 (760.9–818.4)	787.7 (756.5–805.4)	0.017
Normalized cortical grey matter volume (cm^3^)	674.9 (638.1–708.5)	641.1 (613.4–682.9)	<0.001
Normalized ventricular volume (cm^3^)	28.6 (23.0–42.1)	35.9 (24.7–44.1)	0.004
PBVC (%)	N/A	−0.45 (−0.92–−0.02)	N/A
Annualized PBVC rate (%/year)	N/A	−0.13 (−0.46–−0.02)	N/A
T2LL (cm^3^)	5.9 (2.4–10.9)	5.5 (3.3–7.5)	1.000

Values are given as median (25th–75th quartile). Significance (*p* < 0.05) was assessed between subgroups by the paired-samples sign test (*n* = 22). BL = baseline; FU = follow-up; MRI = magnetic resonance imaging; *n* = number of subjects; N/A = not applicable; PBVC = percentage brain volume change; T2LL = T2 lesion load.

## Data Availability

The data presented in this study are available from the corresponding author upon reasonable request.
